# Cyclic GMP in the pig vitreous and retina after experimental retinal detachment

**Published:** 2008-02-04

**Authors:** Roselie M.H. Diederen, Ellen C. La Heij, Marijke A.M. Lemmens, Aize Kijlstra, Jan de Vente, Fred Hendrikse

**Affiliations:** 1 Eye Research Institute Maastricht, Department of Ophthalmology, University Hospital Maastricht, The Netherlands; 2 European Graduate School of Neuroscience (EURON), Psychiatry and Neuropsychology, University of Maastricht, The Netherlands; 3 Animal Sciences Group, Wageningen UR, Lelystad, The Netherlands

## Abstract

**Purpose:**

Earlier studies have revealed a decreased level of cGMP in vitreous fluid obtained from patients with a retinal detachment. To further investigate this phenomenon, we developed an experimental retinal detachment model in pigs.

**Methods:**

Experimental unilateral retinal detachments were induced in pig eyes by subretinal injection of 0.25% sodium hyaluronate. Fourteen days later the vitreous and retinas were analyzed for cGMP expression. Following enucleation, the retinas were incubated in the presence of a nonselective phosphodiesterase inhibitor (IBMX), and the particulate guanylyl cyclase stimulator atrial natriuretic peptide (ANP) or the soluble guanylyl cyclase stimulator sodium nitroprusside (SNP). cGMP was visualized in retinal wholemounts by immunochemistry combined with a computer based stereology system. cGMP levels in vitreous were determined by ELISA.

**Results:**

The mean vitreous cGMP level in pig eyes with a retinal detachment (1.45 pmol/ml) was significantly lower compared to the mean level of cGMP in healthy pig eyes (4.61 pmol/ml; p=0.028 was considered significant). In the inner retina, ANP as well as SNP induced cGMP immunoreactivity in both detached and healthy retinas. After incubation with ANP, cGMP could also be detected in the outer nuclear layer of the detached retina, whereas this was not the case in the normal retina.

**Conclusions:**

Experimental retinal detachment in the pig eye leads to a decrease of cGMP levels in vitreous similar to that observed in clinical studies. This model may be helpful to analyze the mechanisms involved in cGMP dynamics following retinal detachment.

## Introduction

A retinal detachment is defined as a break between the photoreceptor layer and the retinal pigment epithelium (RPE), whereby the formed space is filled with fluid. A retinal detachment can cause serious visual impairment of the affected eye, depending, among other factors, upon the time interval between the initial detachment and the surgical reattachment [1]. Even though surgical repair of the detached retina is often anatomically successful, the affected eye rarely regains its original acuity [2-4]. This may be due to irreversible damage to the retina, caused by hypoxia as well as ischemia of retinal cells, such as the photoreceptor or Müller cells [4,5]. To analyze the damage to photoreceptor cells, we began studies to investigate the level of cyclic guanosine monophosphate (cGMP) in the surrounding fluids [6]. cGMP was chosen as a marker since it is an important molecule in the metabolic cascade of photoreceptor signal transduction. The production of cGMP is catalyzed by guanylyl cyclases (GCs), which are found in photoreceptor cells [7] and inner retinal neurons [8].

Two different GCs catalyze the conversion of the substrate guanosine ‘5-triphosphate into cGMP: particulate (membrane-bound) GC (pGC) and soluble (cytosolic) GC (sGC). sGC is activated by nitric oxide (NO). An increase in retinal tissue levels of NO using the NO donor, sodium nitroprusside (SNP), results in the induction of cGMP immunoreactivity in amacrine, ganglion, and bipolar cells [9,10]. pGCs are either ligand-activated receptors (e.g., natriuretic peptides), or calcium-regulated guanylyl cyclase [10]. In the turtle and rat retina, stimulation with exogenous natriuretic peptides leads to an increase of total retinal cGMP levels [10,11]. All three natriuretic peptides, atrial natriuretic peptide, brain natriuretic peptide, and C-type natriuretic peptide, have been observed in human ganglion and RPE cells [12]. In situ hybridization techniques applied in monkey eyes showed GC transcripts to be localized exclusively along the retinal outer nuclear layer, which corresponded to the nuclei of the rod and cone photoreceptor cells [13]. Studies describing both enzymatic pathways involved in the cGMP production in the rat retina show that the NO-cGMP pathway (via sGC) exists mainly in the inner nuclear layer, whereas the ANP-cGMP pathway (via pGC) is predominantly present in the rod photoreceptors [10,11,14,15]. Cyclic GMP is hydrolyzed to inactive 5′ –derivates by 3′, 5′- cyclic nucleotide phosphodiesterases (PDEs) which have now been divided into 11 different subfamilies (PDE1-PDE11). The major function for PDEs in the cell is to diminish the amplitude of the cyclic nucleotide second messenger signal and to shorten the duration of its action [16].

The increased rate of fluid absorption from the subretinal space indicates a possible role for cGMP in the clearance of subretinal fluid after retinal detachment [16]. The involvement of cGMP during a retinal detachment was furthermore suggested by a previous study, in which we observed a decreased level of cGMP in vitreous and subretinal fluid of patients with retinal detachment [6]. Moreover, cGMP levels decreased further as retinal detachment duration was prolonged.

As yet, no explanations are available to explain the decrease in cGMP concentration after retinal detachment. To be able to address these questions , we found it necessary to develop an experimental animal model. In this study we chose the pig as the experimental animal because the size and the physiology of the pig eye shows great resemblance to the human eye.

## Methods

### Retinal Detachments

Six Dutch domestic female pigs (Dutch landrace; 28- 33 kg in weight; 3 months old) were used as experimental animals (Varkensproefbedrijf, Wageningen UR, Lelystad, The Netherlands).

Unilateral retinal detachments were created in the right eyes of six pigs. Animals were anesthetized by inhalation of nitrous oxide and isoflurane. Via an entry site at the level of the pars plana, between 0.1 and 0.5 ml of a solution of 0.25% sodium hyaluronate (Pharmacia,Uppsala,Sweden) was slowly infused between the neural retina and RPE via a 20-gauge subretinal canula attached to a 5 ml syringe.

During the infusion we visually checked perfusion of the ophthalmic artery to assure that an intraocular pressure rise would not result in arterial occlusion. Healon was used to prevent spontaneous reattachment of the retina. All surgery was done using an operating microscope and using similar equipment for retinal surgery in the (human) clinical operating room as described earlier [17]. After making an incision on the temporal side of the optic disc, 0.25% sodium hyaluronate was infused as described above, to create a retinal detachment from the temporal side of the disk up to the midperiphery of the retina, so that approximately one -third to half of the whole retina was detached.

Approximately half the retina was detached in each eye. By indirect ophthalmoscopy, we made sure that the retinas remained detached for the duration of the experiment. Following surgery, the entry port was sutured closed, and animals were given two times 250 mg acetazolamide daily for the next three days to prevent intraocular pressure rise. The left eyes were used as the control for all the experiments. Following 14 days of detachment, the animals were euthanized using intravenous pentobarbital (200 mg/ml) (Euthesate; Aesculaap, Boxtel, The Netherlands) and both eyes were enucleated.

A period of 14 days was chosen since this was also a time period whereby human patients showed a marked decrease in their intraocular cGMP levels [6]. All experimental procedures conformed to the ARVO statement for the Use of Animals in Ophthalmic and Vision Research and the guidelines of the Animal Resource Center of the University of California Santa Barbara (Santa Barbara, CA).

**Figure 1:**
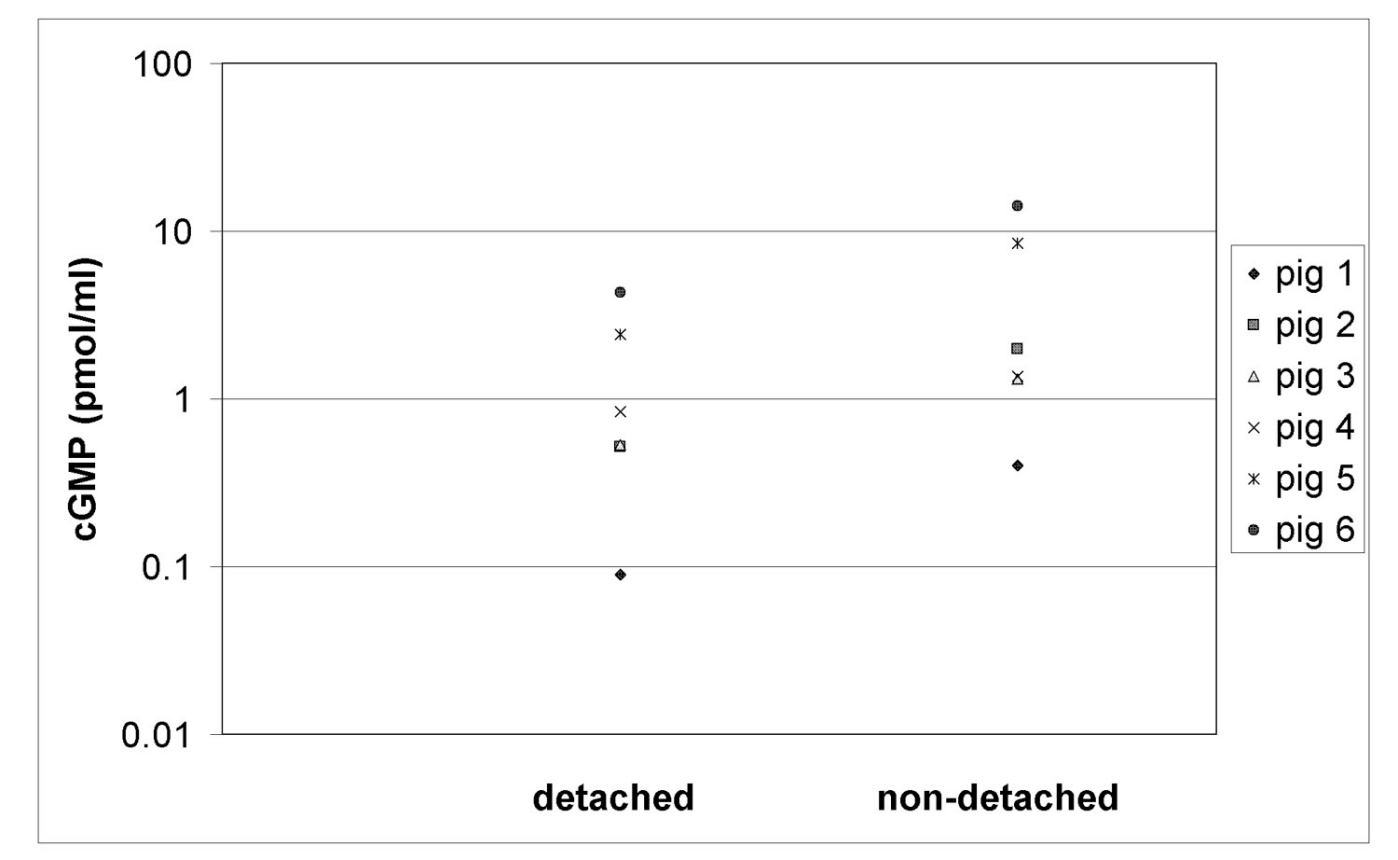
Effect of retinal detachment on vitreous cyclic guanosine monophosphate (cGMP)(pmol/ml) levels. A retinal detachment was induced in the right eyes of six pigs. Fourteen days later the eyes were enucleated and vitreous from detached and non-detached eyes was collected and analyzed for cGMP content with an immunoassay. The figure shows the individual cyclic GMP levels in the eyes with a detached or non-detached retina from each pig. It is clear that the vitreous cGMP level in the eyes with a detached retina is lower that that from the control eyes.

### Vitreous fluid collection

Vitreous fluid was obtained from both eyes of six pigs, two weeks post experimental retinal detachment. Within a few minutes after enucleation, approximately 1.5 ml of vitreous fluid was aspirated from the intact eye, using a syringe fitted with a 20 gauge needle. The vitreous sample was immediately centrifuged, and the supernatant was stored at −80 °C until tested for cGMP.

### Cyclic GMP assay

Extracellular cGMP was measured in duplicate using a commercially available ELISA kit (Assay Designs, Ann Arbor, MI). We used 100 μl of undiluted vitreous fluid and performed the measurement according to the manufacturer’s instructions. The amount of cGMP was expressed as pmol/ml of undiluted vitreous. The sensitivity of the cGMP assay was 0.088 pmol/ml.

### Tissue Preparation

After enucleation and vitreous sampling, an incision was made at the margin of the ora serrata to remove the lens and ciliary body. Subsequently the retina was removed, and randomly chosen small pieces were incubated for 40 min in aerated Krebs buffer (pH 7.4) with or without 1 mM IBMX, a nonspecific PDE inhibitor (Sigma, Amsterdam, The Netherlands). During the last ten minutes of the incubation, the tissue was stimulated with 100 μM of the NO donor SNP or 0.1 μM of the pGC stimulator ANP.

### Immunohistochemistry

To determine the site of cGMP production in the pig retina the following experiments were performed. As mentioned in the previous paragraph, pieces of retinal tissue from eyes with and without a retinal detachment were incubated in vitro, and cGMP production was induced by stimulation of the sGCs (by SNP) or pGCs (by ANP) with a simultaneous inhibition of cGMP breakdown by PDEs using IBMX. After incubation the retinal tissue pieces were fixed with 4% freshly depolymerized cold (4 °C) paraformaldehyde. Retina wholemounts were washed 3 times for 5 min, twice with Tris-buffered saline (TBS) and once with TBS containing 0.3% Triton X-100 (TBS-T). Samples were subsequently incubated overnight at 4 °C with the primary antibodies. Cyclic GMP was visualized using sheep antiformaldehyde-fixed cGMP antiserum diluted 1:4000 in TBS-T as we described earlier [18].

The following day, the samples were washed for 15 min with TBS first, then TBS-T and TBS. The tissues were subsequently incubated with Alexa 488 conjugated donkey antisheep IgG (Molecular Probes, Breda, The Netherlands), diluted 1:100 in TBS-T, for 1.5 h at room temperature. Negative controls were processed in exactly the same way with the omission of the primary antibody. The tissues were washed three more washings in TBS, and the nuclei of the cells were stained with Hoechst 33342, (Sigma, Zwijndrecht, The Netherlands) for 20 min at room temperature (diluted 1:1000) and finally washed again in TBS. Wholemounts were coverslipped with TBS:glycerol (1:2 v/v).

**Figure 2:**
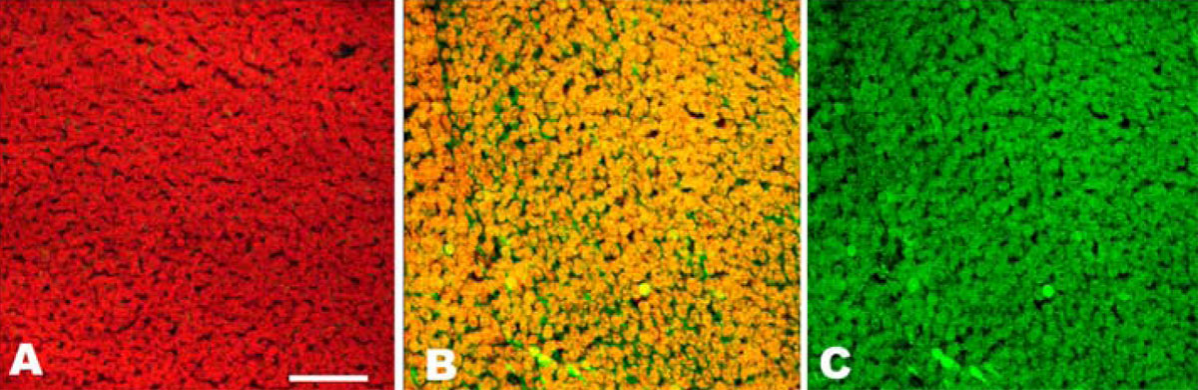
In vitro expression of cyclic guanosine monophosphate (cGMP) in the photoreceptor layer of a normal or detached pig retina. Small pieces of retina were incubated with IBMX (a non-specific PDE inhibitor) and the particulate guanylyl cyclase (pGC) stimulator atrial natriuretic peptide (ANP). The expression of cGMP (cGMP: green color) in the retina was then analyzed by immunohistochemistry and nuclei were counterstained with Hoechst 33342 (red). Under these conditions no cGMP signal was visible in the photoreceptor layer of the healthy retina (Panel A). The detached retina on the other hand displayed strong cGMP expression in the photoreceptor layer (Panels B and C). Panel B is a double staining exposure for the nuclei and cGMP, showing the presence of cGMP in the nuclei of the cells (yellow) and in the cytoplasma (green). In Panel C we only show the green signal, showing strong cGMP immunoreactivity in the same piece of retina. Scale bar represents 25 μm.

### Microscopy

Retinal wholemounts were analyzed by fluorescence microscopy using two-photon laser scanning microscopy (TPLSM). TPLSM was performed as previously described using a microscope objective (40x; water dipping; numerical aperture [NA]=1.0) connected to an upright Nikon E600FN microscope (Nikon Corporation, Tokyo, Japan) [19]. Further magnification, when needed, was achieved by an optical zoom in the scan head. To remove background noise, we filtered each image using the Kalman filtering procedure on three subsequent images during experiments. The fluorescent secondary antibody Alexa 488 was mainly visible in the green channel, whereas Hoechst was only visible in the red channel. The obtained images (coded green and red, respectively) were combined into single images as needed. Images were taken with a MBF Bioscience Stereo Investigator Confocal Spinning Disk (SI-SD) system (MBF Bioscience, Williston, VT), consisting of a modified Olympus BX51 fluorescence microscope (Olympus, Tokyo, Japan) with UPlanSApo objectives, customized spinning disk unit (DSU; Olympus, Zoeterwoude, The Netherlands), computer-controlled excitation and emission filter wheels (Olympus), three-axis high-accuracy computer-controlled stepping motor specimen stage (4x4 Grid Encoded Stage; Ludl Electronic Products, Hawthorne, NY), linear z-axis position encoder (Ludl), ultra-high sensitivity monochrome electron multiplier CCD camera (1,000x1,000 pixels, C9100–02; Hamamatsu Photonics, Hamamatsu City, Japan) and controlling software (MBF Bioscience). Pictures were taken in a single focal plane with a 40x UPlanSApo objective (N.A.=0.9) and processed with Imaris software (Version 4.0; Bitplane, Zurich, Switzerland). Only minor adjustments of contrast and brightness were made without altering the appearance of the original materials. No deconvolution was performed on the images. The computer-based stereology system that was used allows encoding of the Z-axis position thereby measuring the actual focus position within the retina.

### Statistical analysis

**Figure 3:**
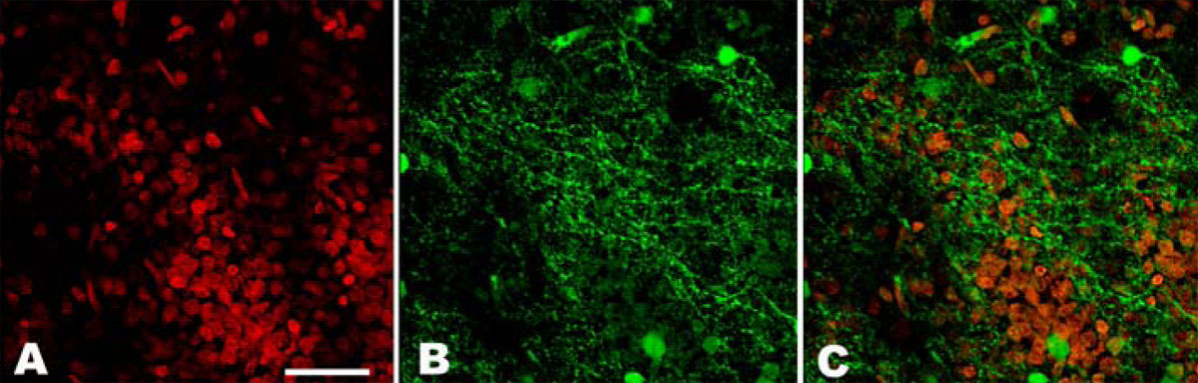
In vitro expression of cyclic guanosine monophosphate (cGMP) in the outer plexiform layer of a detached retina. Small pieces of retina were incubated with IBMX (a non-specific PDE inhibitor) and the particulate guanylyl cyclase (pGC) stimulator atrial natriuretic peptide (ANP). The expression of cGMP (cGMP: green color) in the retina was then analyzed by immunohistochemistry and nuclei were counterstained with Hoechst 33342 (red). Following stimulation with ANP in the presence of IBMX, both the detached and healthy retina showed cGMP-immunolabeling in the outer plexiform layer. Panel A shows the labeling of the outer plexiform layer of a detached retina with Hoechst. Panel B shows the cGMP stain (green) of the outer plexiform layer probably representing photoreceptor axons and dendrites. Panel C is a double staining exposure of the nuclei and cGMP. Scale bar represents 20 μm.

Because the cGMP values were paired and nonparametric, we used the Wilcoxon signed ranks test to compare the cGMP content in pig vitreous fluid of eyes with and without retinal detachment. Differences were considered significant when the p value was less than 0.05 (two-tailed).

## Results

We were able to detect cGMP in all the vitreous fluid samples obtained from the pig eyes used in this study. The mean vitreous level of cGMP was 1.45 pmol/ml (range: 0.09 to 4.32 pmol/ml) in eyes with retinal detachment. In the control (contralateral) eyes, the mean vitreous level of cGMP was 4.61 pmol/ml (range between 0.40 to 14.10 pmol/ml). The difference in vitreous cGMP levels between eyes with detached retinas and control eyes was significant (p=0.028 by Wilcoxon signed ranks test). A large variation in cGMP levels was observed between pigs. In each pig, however, the cGMP level in the right eye with a retinal detachment was always lower (between 1.6 and 5.8 times) than in the healthy left eye (Figure 1).

### Immunohistochemistry

**Figure 4:**
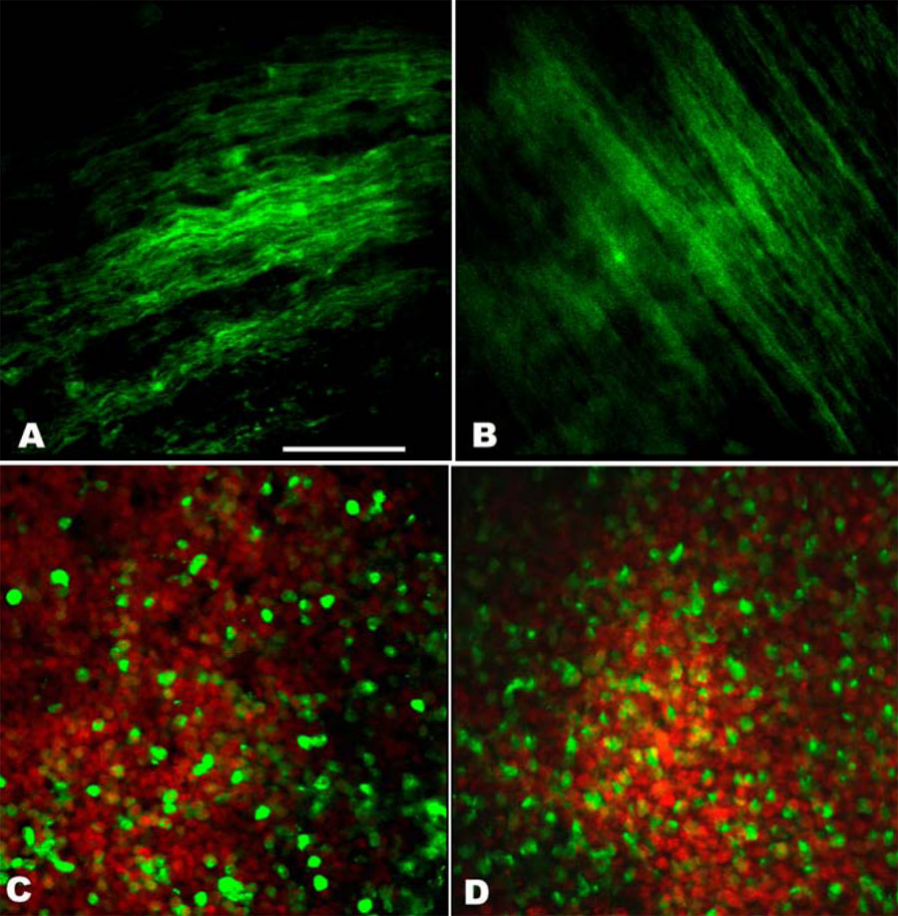
In vitro expression of c cyclic guanosine monophosphate (cGMP) in the nerve fiber layer and inner nuclear layer of a healthy or detached retina. Small pieces of retina were incubated with IBMX (a non-specific PDE inhibitor) and the soluble guanylyl cyclase (sGC) stimulator sodium nitroprusside (SNP). The expression of cyclic GMP (cGMP: green color) in the retina was analyzed by immunohistochemistry and nuclei were counterstained with Hoechst 33342 (red). Panels A and B show a similar cGMP expression in nerve fiber layer of the normal (A) or detached (B) retina. Panel C (normal retina) and Panel D (detached retina) show cGMP staining in the inner nuclear cells stained in red. Scale bar represents 50 μm.

The expression of cGMP in the retina was analyzed by immunohistochemistry. No cGMP expression could be observed in any of the retinal cells (either detached or healthy) if phosphodieasterase activity had not been blocked with IBMX. This indicates that under normal conditions, cGMP has an extremely short half life. In the absence of cyclase stimulation but in the presence of PDE blockers, we observed cGMP immunoreactivity in the inner nuclear layer of the detached as well as the healthy retinas. Addition of 0.1 μM ANP as a stimulator of pGC, combined with PDE inhibition, resulted in the appearance of a strong cGMP signal in both the ganglion cells and the inner nuclear layer of the healthy retina. No cGMP signal was visible in the outer nuclear layer of the healthy retina (Figure 2A). The detached retina on the other hand displayed strong cGMP expression not only in the ganglion cells and the inner nuclear layer but also in the outer nuclear layer (Figure 2B,C). Figure 2B shows both the nuclei of the cells (Hoechst staining; red signal) and cGMP (green signal) staining of the outer nuclear layer of a detached retina. Figure 2C only presents the green signal, showing strong cGMP immunoreactivity in the same piece of retina. Following stimulation with ANP in the presence of IBMX, both the control eye and eye with the detached retina showed cGMP-immunolabeling in the inner and outer plexiform layer. Figure 3 presents the labeling of the outer plexiform layer of a detached retina with Hoechst (Figure 3A) and cGMP (Figure 3B) separately and together (Figure 3C) in the same piece of retina. Incubating the retinas in the presence of 100 μM SNP, as a stimulator of sGC in combination with PDE inhibition (with IBMX) resulted in cGMP-immunoreactivity in the ganglion cells, the inner nuclear cell layer (Figure 4 C,D) and the nerve fiber layer of the detached- and healthy retinas (Figure 4A,B). Stimulation with SNP (+ IBMX) did not result in the appearance of cGMP immunoreactivity in the photoreceptor cells of the detached or healthy retina. Incubation of retinas with ANP or SNP (+ IBMX) both resulted in cGMP-immunoreactivity in the inner nuclear layer, although not in all cell types (Figure 4C,D). The aforedescribed experiments thus only showed a difference in cGMP expression between the retinas with and without experimental detachment following in vitro stimulation of pGC. In vitro, we demonstrated that ANP induced synthesis of cGMP in the outer nuclear layer of the detached retina, whereas this was not the case in the attached retina.

## Discussion

In the current study, we demonstrated the presence of cGMP in pig vitreous fluid and showed a significantly lower vitreous cGMP level following retinal detachment. The animal model thus confirms earlier findings in humans in which we showed a significantly lower vitreous cGMP concentration in patients with an rhegmatogenous retinal detachment as compared to controls [6]. Our findings are also in accordance with earlier findings in a rabbit model that showed that vitreous cGMP levels were almost 60% lower in eyes with a retinal detachment than in control eyes [20].

Cyclic GMP production is catalyzed by either sGC, activated by NO or pGC stimulated by natriuretic peptides. After retinal detachment, the activity of both sGC and pGC could be reduced, since certain populations of retinal cells, such as the photoreceptor or Mϋller cells, may have undergone degenerative changes induced by hypoxic or ischemic alterations following the detachment [4,5]. A decreased production of cGMP by retinal cells may concurrently lead to a lower release into the extracellular space--in this case, vitreous fluid. This explanation is speculative, however, since no evidence is available to date concerning extracellular transport of cGMP from retinal cells. Previously we showed that in vitro cultured RPE cells can be triggered to produce and secrete cGMP into the culture medium [21]. A similar approach could be used with retinal sections from healthy and detached retinas to assess a possible contribution of sensory retinal cells to the level of cGMP in vitreous or subretinal fluids.

We measured cGMP in vitreous fluid and retinal sections using immunoassays. The presence of cGMP immunoreactivity is considered to be equivalent to a potential biologic activity of the cGMP molecule as we recently showed in a model of rat aorta contraction and dilation [22]. Biologic activity of cGMP depends on its targets, which include the cGMP-dependent protein kinases, cyclic nucleotide-gated channels, cAMP-dependent protein kinase, and PDEs [23]. The actual presence of these targets may differ among cells, explaining the wide variety of activities of cGMP.

The cellular concentration of cGMP represents the net balance between synthesis by guanylyl cyclases and breakdown into 5′-cGMP by cyclic nucleotide PDEs [24]. The decreased cGMP concentration in the vitreous after retinal detachment could also be the result of increased PDE activity. This seems less plausible, however, since no detectable PDE activity seems to be present in vitreous fluid [6]. After rhegmatogenous retinal detachment, vitreous passes through a retinal break into the subretinal space and then comes into direct contact with the RPE layer. It is possible that the cGMP in the vitreous is degraded by PDEs located in the RPE cells. This may be plausible since earlier studies have shown the presence of at least PDE 2, PDE 5, and PDE 9 in RPE cells [25].

Stimulation of sGC by the NO donor SNP resulted in cGMP expression in the inner pig retina, especially in the ganglion cells and bipolar cell layer. Further studies are needed to determine the pathways that are targeted by cGMP within these cells.

The effect of SNP on cGMP immunoreactivity in the pig retina was similar to findings reported in other species [9,10]. After stimulation of the particulate guanylyl cyclase with ANP, cGMP expression was observed in the outer nuclear layer of the eyes with a detached retina. Under similar conditions the outer nuclear layer of the eyes with a normal attached retina did not express cGMP. The explanation for this difference remains speculative. It may be caused by changes in local PDE levels or by changes in the ANP receptor expression of cells in the outer retina. Further analysis on retinal PDE expression following detachment is necessary to fully understand the dynamics of cGMP in the retina.

The increased cGMP expression in the detached retina compared with the normal retina seems to contradict the finding that cGMP levels were lower in the vitreous of eyes with a detached retina. On the other hand this could also be explained by assuming a decreased secretion of cGMP by the retinal cells leading to lower levels in the extracellular space and concomitant intracellular accumulation.

Most studies concerning retinal cGMP have focused on its role in phototransduction [26]. Little is known about the changes in cGMP expression in the retina and the vitreous after retinal detachment. It has been shown that cGMP stimulates subretinal fluid absorption whereas cAMP inhibits this process [27]. Until now, we have only measured cGMP in ocular fluids following clinical or experimental detachments. Simultaneous determination of cAMP may be useful for the understanding of subretinal fluid dynamics after the formation of retinal break.

## References

[1] Diederen RM, La Heij EC, Kessels AG, Goezinne F, Liem AT, Hendrikse F (2007). Scleral buckling surgery after macula-off retinal detachment: worse visual outcome after more than 6 days.. Ophthalmology.

[2] Burton TC (1982). Recovery of visual acuity after retinal detachment involving the macula.. Trans Am Ophthalmol Soc.

[3] Sabates NR, Sabates FN, Sabates R, Ledd KY, Ziemianski MC (1989). Macular changes after retinal detachment surgery.. Am J Ophthalmol.

[4] Mervin K, Valter K, Maslim J, Lewis G, Fisher S, Stone J (1999). Limiting photoreceptor death and deconstruction during experimental retinal detachment: the value of oxygen supplementation.. Am J Ophthalmol.

[5] Lewis G, Mervin K, Valter K, Maslim J, Kappel PJ, Stone J, Fisher S (1999). Limiting the proliferation and reactivity of retinal Muller cells during experimental retinal detachment: the value of oxygen supplementation.. Am J Ophthalmol.

[6] La Heij EC, Blaauwgeers HG, de Vente J, de Vente J, Markerink M, Liem AT, Kessels AG, Steinbusch H, Hendrikse F (2003). Decreased levels of cGMP in vitreous and subretinal fluid from eyes with retinal detachment.. Br J Ophthalmol.

[7] Schraermeyer U, Esser P, Grisanti S, Rack M, Heimann K (1997). Cytochemical localization of guanylate cyclase in photoreceptor cells of the mouse.. Graefes Arch Clin Exp Ophthalmol.

[8] Blute TA, Lee HK, Huffmaster T, Haverkamp S, Eldred WD (2000). Localization of natruretic peptides and their activation of particulate guanylate cyclase and nitric oxide synthase in the retina.. J Comp Neurol.

[9] Gotzes S, de Vente J, Muller F (1998). Nitric oxide modulates cGMP levels in neurons of the inner and outer retina in opposite ways.. Vis Neurosci.

[10] Blute TA, Velasco P, Eldred WD (1998). Functional localization of soluble guanylate cyclase in turtle retina: modulation of cGMP by nitric oxide donors.. Vis Neurosci.

[11] Mikami Y, Hara M, Yasukura T, Uyama M, Minato A, Inagaki C (1995). Atrial natriuretic peptide stimulates Cl- transport in retinal pigment epithelial cells.. Curr Eye Res.

[12] Rollin R, Mediero A, Roldan-Pallares M, Fernandez-Cruz A, Fernandez-Durango R (2004). Natriuretic peptide system in the human retina.. Mol Vis.

[13] Shyjan AW, de Sauvage FJ, Gillett NA, Goeddel DV, Lowe DG (1992). Molecular cloning of a retina-specific membrane guanylyl cyclase.. Neuron.

[14] Mills SL, Massey SC (1995). Differential properties of two gap junctional pathways made by AII amacrine cells.. Nature.

[15] Ahmad I, Barnstable CJ (1993). Differential laminar expression of particulate and soluble guanylate cyclase genes in rat retina.. Exp Eye Res.

[16] Beavo JA (1995). Cyclic nucleotide phosphodiesterases. functional implications of multiple isoforms.. Physiol Rev.

[17] La Heij EC, Hendrikse F, Kessels AGH (2001). Results and complications of temporary silicone oil tamponade in patients with complicated retinal detachments.. Retina.

[18] De Vente J, Markerink-van Ittersum M, van Abeelen J, Emson PC, Axer H, Steinbusch HW (2000). NO-mediated cGMP synthesis in cholinergic neurons in the rat forebrain: effects of lesioning dopaminergic or serotonergic pathways on nNOS and cGMP synthesis.. Eur J Neurosci.

[19] Van Zandvoort M, Engels W, Douma K, Beckers L, Oude Egbrink M, Daemen M, Slaaf DW (2004). Two-photon microscopy for imaging of the (atherosclerotic) vascular wall: a proof of concept study.. J Vasc Res.

[20] Dalma-Weiszhauz J, Blumenkranz M, Hartzer M, Hernandez E (1993). Intraocular extracellular cyclic nucleotide concentrations: the influence of vitreous surgery.. Graefes Arch Clin Exp Ophthalmol.

[21] Diederen RMH, La Heij EC, Markerink-van Ittersum M, Kijlstra A, Hendrikse F, de Vente J (2007). Cyclic GMP synthesis by human retinal pigment epithelial cells is mainly mediated via the particulate guanylyl cyclase pathway.. Ophthalmic Res.

[22] Rietjens SJ, Bast A, de Vente J, Haenen GR (2007). The olive oil antioxidant hydroxytyrosol efficiently protects against the oxidative stress-induced impairment of the NObullet response of isolated rat aorta.. Am J Physiol Heart Circ Physiol.

[23] Rehmann H, Wittinghofer A, Bos JL (2007). Capturing cyclic nucleotides in action: snapshots from crystallographic studies.. Nat Rev Mol Cell Biol.

[24] Francis SH, Corbin JD (1999). Cyclic nucleotide-dependent protein kinases: intracellular receptors for cAMP and cGMP action.. Crit Rev Clin Lab Sci.

[25] Diederen RMH, La Heij EC, Markerink-van Ittersum M, Kijlstra A, Hendrikse F, de Vente J (2006). Selective blockade of phosphodiesterases type 2, 5 and 9 results in cGMP accumulation in retinal pigment epithelium cells.. Br J Ophthalmol.

[26] Baylor DA (1987). Photoreceptor signals and vision.. Invest Ophthalmol Vis Sci.

[27] Marmor MF, Negi A (1986). Pharmacologic modification of subretinal fluid absorption in the rabbit eye.. Arch Ophthalmol.

